# Variations in species diversity patterns and community assembly rules among vegetation types in the karst landscape

**DOI:** 10.3389/fpls.2024.1338596

**Published:** 2024-02-22

**Authors:** Longchenxi Meng, Yong Li, Luyao Chen, Mingzhen Sui, Guangqi Zhang, Qingfu Liu, Danmei Chen, Yuhang Wu, Zeyu Yang, Shiren Chen, Rui Yang, Lipeng Zang

**Affiliations:** ^1^ Research Center of Forest Ecology, College of Forestry, Guizhou University, Guiyang, China; ^2^ Guizhou Libo Karst Forest Ecosystem National Observation and Research Station, National Forestry and Grassland Administration, Libo, China

**Keywords:** vegetation types, community assembly, karst landscape, species diversity, species abundance distribution

## Abstract

The various vegetation types in the karst landscape have been considered the results of heterogeneous habitats. However, the lack of a comprehensive understanding of regional biodiversity patterns and the underlying ecological processes limits further research on ecological management. This study established forest dynamic plots (FDPs) of the dominant vegetation types (shrubland, SL; mixed tree and shrub forest, MTSF; coniferous forest, CF; coniferous broadleaf mixed forest, CBMF; and broadleaf forest, BF) in the karst landscape and quantified the species diversity patterns and potential ecological processes. The results showed that in terms of diversity patterns, the evenness and species richness of the CF community were significantly lower than other vegetation types, while the BF community had the highest species richness. The other three vegetation types showed no significant variation in species richness and evenness. However, when controlling the number of individuals of FDPs, the rarefied species richness showed significant differences and ranked as BF > SL > MTSF > CBMF > CF, highlighting the importance of considering the impacts of abundance. Additionally, the community assembly of climax communities (CF or BF) was dominated by stochastic processes such as species dispersal or species formation, whereas deterministic processes (habitat filtering) dominated the secondary forests (SL, MTSF, and CBMF). These findings proved that community assembly differs mainly between the climax community and other communities. Hence, it is crucial to consider the biodiversity and of the potential underlying ecological processes together when studying regional ecology and management, particularly in heterogeneous ecosystems.

## 1 Introduction

As the global species extinction rate increases with habitat degradation, maintaining biodiversity has become a severe ecological issue, especially in extreme ecosystems (Butchart et al., 2010; Johnson et al., 2017). Previous studies suggested that biodiversity reflects the extent of the history of life on Earth and serves as an essential foundation for ecosystem sustainability (GE, 2017; Mi et al., 2021). The four levels of biodiversity include gene, species, ecosystem, and landscape, and species diversity is crucial for biodiversity conservation as it directly reflects the community structure, stability, and development stage (Wang et al., 2022). Numerous studies have explored large-scale species richness variations along elevation or latitude (Chen et al., 2015; Fontana et al., 2020). However, the regional diversity patterns and driving mechanisms among vegetation types are unclear, thus requiring academic attention.

A growing body of studies showed that species richness depends heavily on abundance (Xue et al., 2021; Feng et al., 2022), emphasizing the importance of considering the confusing impacts of abundance accumulation. However, researchers prefer to correct rather than quantifying confusing impacts of sample sizes. Consequently, the current challenges under confusing impacts of sample sizes leads to a misunderstanding of species diversity patterns in ecological researches (Feng et al., 2022). Despite its intuitiveness and universality, species richness is oversimplifying as it is determined by combining the total numbers of species (Chao et al., 2014), and it increases nonlinearly with the increasing sampling size (Chase et al., 2018). Therefore, in addition to the traditional richness index, more convictive indices such as the rarefied species richness should be employed to address the impact of varying sample sizes when exploring species diversity pattern differences (Roswell et al., 2021). Although species richness and species evenness are both important components of species diversity, previous studies focused mainly on species richness while seldom considering species evenness (Huang et al., 2015; Villa et al., 2018). Since the mono diversity index can not account for various community components and almost all ecological factors or processes in a community can potentially affect species abundance or regulate species relative abundances (Feng et al., 2021), the species diversity patterns must be explored based on comprehensive, multi-index measurements rather than a single component.

Species abundance distribution (SAD) effectively reflects the species richness and evenness as it directly quantifies the species and its dominance in the community (McGill et al., 2007; Baldridge et al., 2016). However, the multiple dimensions and components of biodiversity render it difficult to identify the model fitting the SADs best and, in turn, reveal species diversity patterns (Feng et al., 2022). In addition to revealing the biodiversity patterns, determining the ecological process driving the observed biodiversity patterns is another crucial unsolved issue. Traditional niche theory holds that species coexist and maintain diversity over time only if specific factors, such as niche differentiation, prevail during community construction (Haegeman and Loreau, 2011). However, this theory faces a vital challenge in explaining why so many species with similar niche spaces coexist at the local scale. Neutral theory proposed by Hubbell (2001) suggests that species exhibit similar population dynamics due to shared characteristics. Stochastic processes control community construction, including birth, death, colonization, and speciation (Zhou and Ning, 2017). Numerous studies indicated that both deterministic and stochastic processes contributed to community construction (Vilmi et al., 2021). Various deterministic and stochastic processes acting across scales form the actual community (Yuan et al., 2011; Su et al., 2023). Normally, species diversity pattern variations are primarily affected by dispersal limitations, local species pools, habitat filtering, and species interactions at local scales (Sabatini et al., 2022). However, the processes maintaining the species diversity patterns and the extent are still under debate. Previous research indicated that species dispersal limitations and habitat filtering played distinct roles in the plant community across the environmental gradients (Flinn et al., 2010). Sperry et al. (2019) found that species migrated from high-diversity areas to low-diversity areas, and species with greater dispersal abilities were more likely to spread from high-diversity areas to low-diversity areas. A study by Parra-Tabla et al. (2018) concluded that habitat filtering constitute a major driver of plant diversity and species composition.

Constructing suitable models to fit SAD curves can well reflect various community structures and the underlying ecological processes (Tan et al., 2020). For example, the most common lognormal and logseries methods depict different scenarios of the relative abundance of species relative to the size and structure of an assemblage (Magurran, 2021). The logseries distribution derives from Poisson sampling of a gamma distribution, while the lognormal distribution represents a situation in which the logarithms of the different species’ abundances follow a Gaussian distribution (Matthews and Whittaker, 2014b). Previous studies suggested three steps for fitting and evaluating SAD models: 1) fitting the model and estimating the parameters; 2) determining the goodness of fit of the model; and 3) comparing the goodness of fit of the model with that of other models (Matthews and Whittaker, 2014a). In recent years, many studies have attempted to describe the shape of the SAD distribution, and quantifying the shape of SAD facilitates the detection of SAD differences and the identification of their drivers (Baldridge et al., 2016; Feng et al., 2021). For example, Yin et al. (2018) found that the SADs of the log Cauchy model could better reveal the inherent features of community structure and dynamics. Qiao et al. (2015) fitted a non-neutral model of community assembly to these SADs, calculated the fitted deviation from neutrality, and observed the correlations between the fitted deviation from neutrality and geographical and environmental variations. The neutral community model (NCM) has been considered a convictive local-scale model to predict the SADs and reveal the ecological processes (Matthews and Whittaker, 2014a). By comparing the actual SAD with the zero-sum multinomial distribution, a most important hypothesis of neutral theory, the dominant ecological process can be determined. However, NCM allows species to have a competitive advantage or disadvantage, and the nearly neutral model transforms the model into a continuous form (Sloan et al., 2006; Chen et al., 2019).

Karst landform is an exceptionally distinctive geological phenomenon, rising to vegetation types that are entirely distinct from subtropical evergreen broadleaf forests at the same latitudes, normally characterized by subtropical mixed evergreen and deciduous broad-leaved forests (Dai et al., 2023). Due to shallow soil layers and poor water retention and fertility capabilities, the karst forest is vulnerable to various anthropogenic disturbances such as logging, commercial exploitation and shifting cultivation (Wang et al., 2023). During the last century, it facing various degradation to shrubland or other vegetation types, even degradation of rocky desertification, with biodiversity lost and ecosystem functioning disordered (Yao et al., 2023). Balancing environmental quality and societal development has been a fundamental issue for regional environmental management. Previous studies have focused on soil properties and microbial community structure under various vegetation types (Yan et al., 2020; Guan et al., 2022; Lu et al., 2022). However, as the basis for understanding regional environment management, the plant biodiversity pattern and its underlying ecological processes among various vegetation types have received less consideration, which should be the scientific basis of regional environmental management. Thus, by establishing FDPs in dominant vegetation types and quantifying the species biodiversity patterns, this study aimed to solve the following questions: (1) How do species diversity patterns vary among vegetation types in karst landscape? (2) What process determines the variations in diversity patterns?

## 2 Materials and methods

### 2.1 Study area and data collection

**Figure 1:**
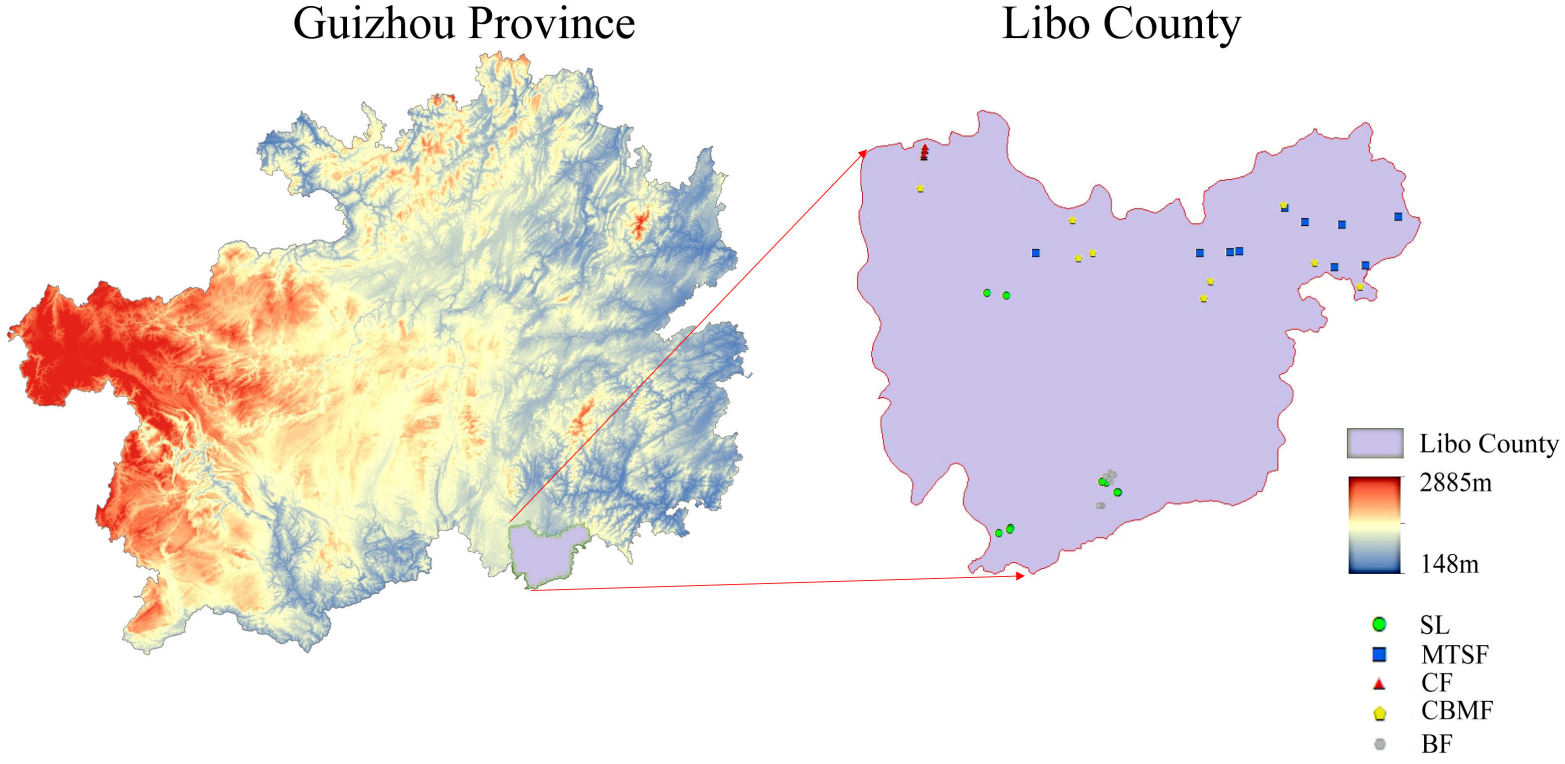
Geographic location and sampling site of the study areas. SL, shrubland; MTSF, mixed tree and shrub forest; CF, coniferous forest; CBMF, coniferous broadleaf mixed forest; BF, broadleaf forest.

This study was conducted at Maolan National Nature Reserve (107°52’E to 108°05’E and 25°09’N to 25°20’N) (**Figure 1**), with a total area of 21,285 hectares. It is the only remaining, original, stable karst forest system at the same latitude worldwide, with a mean elevation of 800 m. The rocks in the region consist predominantly of pure carbonate limestone and dolomite, while the soil is primarily weakly alkaline black lime soil. The region has an average annual temperature of 15.3°C, averaging 5.2°C in January and 23.5°C in July. The growing period spans 237 days, while the frost-free period totals 10 days. The annual precipitation is 1752 mm, typically from May to October. Additionally, the annual average relative humidity is 83% (Zhang et al., 2012). The most widely distributed vegetation types in the nature reserve include SL, MTSF, CF, CBMF, and BF. The SL communities are mainly dominated by *Cornus parviflora* and *Celtis sinensis*, while *Liquidambar formosana* and *Castanopsis fargesii* were the dominant species in CF. *Pinus kwangtungensis* was the constructive species in the CF, and *Pinus massoniana* was the dominant species in CBMF. In addition, *Acer wangchii* and *Boniodendron minus* were the dominant species in BF.

According to the standard raised by Condit (1998) for the standard handbook of forest dynamic plots (FDPs) establishment, we established 9 to 10 FDPs for each of five dominant vegetation types: shrubland (SL), mixed tree and shrub forest (MTSF), coniferous forest (CF), coniferous broadleaf mixed forest (CBMF), and broadleaf forest (BF) (**Table 1**). The distance between every two plots is greater than 50 m to avoid the confusing impacts of spatial autocorrelation and ecotone and all the woody individuals with DBH > 1 cm in the FDPs were tagged, measured, and identified to species (Condit, 1998).

**Table 1:** Basic information of the established plots: shrubland (SL), mixed tree and shrub forest (MTSF), coniferous forest (CF), coniferous broadleaf mixed forest (CBMF), and broadleaf forest (BF).

Vegetation types	Number	Area (m^2^)	Elevation(m)	Slope	Latitude	Longitude
SL	10	100	762-866	10°-30°	25°09′55″-25°26′19″	107°46′56″-107°56′19″
MTSF	10	400	334-870	5°-45°	25°28′02″-25°32′06″	107°50′26″-108°16′30″
CF	9	400	851-950	20°-60°	25°35′35″-25°36′16″	107°42′21″-107°42′31″
CBMF	9	400	400-799	10°-35°	25°25′57″-25°33′25″	107°42′10″-108°13′47″
BF	10	400	743-840	7°-32°	25°11′46″-25°14′03″	107°54′55″-107°56′02″

### 2.2 Statistical approaches

#### 2.2.1 Variations in species α-diversity patterns among vegetation types

In this study, α-diversity was quantified based on Margalef’s index (Equation 1) and Pielou’s index (Equation 2) (Margalef, 1957; Levins, 1970). To eliminate the influence of sample size on the diversity index, the Rarefied species richness index (Equation 3) (Rarefied SR) was used as an auxiliary parameter for diversity comparison (Hurlbert, 1971). One-way ANOVA was adopted to reveal the significance of the differences among vegetation types. The specific formula is as follows:


(1) Margalef's index =(S−1)lnN

(2) Pielou′s index=HlnS

(3) $$Rarefied\;SR=\sum_{i=1}^{S}\left(1-\frac{\left(\frac{N-x_{i}}{n}\right)}{\frac{N}{n}}\right)$$

where *S* is the number of species, *N* is the total number of individuals of all species, and *n* is the size of samples taken during the sparsity process. In this study, *n* = 10, *x_i_* is the abundance of species *i*, $H=\sum_{i=1}^{s}P_{i}\left(lnP_{i}\right)$ , and *P_i_* is the relative abundance of species *i*.

The plant importance value (IV) index (Equation 4) is calculated with the following formulas (Skeen, 1973):


(4) $$\text{IV}=\left(R_{f}＋R_{d}＋R_{do}\right)/3$$

In the formula $R_{f}=\frac{F_{s}}{T_{f}}$ , *R_f_* is the relative frequency, *F_s_* is the frequency of species occurrence, and *T_f_* is the total frequency of all species. In $R_{d}=\frac{D_{s}}{T_{s}}$ , *R_d_* is the relative density, *D_s_* is the species density, and *T_s_* is the total species density. In the formula $R_{do}=\frac{D_{os}}{T_{d}}$ , *R_do_* is the relative significance, *D_os_* is the species significance, and *T_d_* is the total significance of all species.

Community composition was evaluated by applying a *Bray-Curtis* dissimilarity index to an untransformed abundance matrix for each vegetation types, and then a nonmetric multidimensional scaling method (NMDS) using the ‘metaMDS’ function of the ‘vegan’ package in R statistical software was adopted to illustrated results (Murray-Stoker and Murray-Stoker, 2020). The function has a stable solution with several random starts and standardizes the scaling in the results through principal component rotation. In this way, the variance of the points along the first axis is maximized so that the configurations are easier to interpret. Whether the graph accurately reflects the actual data ordering distribution can be determined based on the stress values. A stress value closer to 0 indicates a better dimensionality reduction effect. The number of dimensions was set to 2 to minimize the stress (Norden et al., 2009).

#### 2.2.2 Variation in first-order community assembly among vegetation types

The SAD curves usually represent the species richness and evenness. The horizontal axis is ranking of species number, while the vertical axis is the actual abundance value. Species richness denotes the number of varied species in the diagram, whereas species evenness is represented by the slope of the curve (Baldridge et al., 2016). The species accumulation curve reflects the cumulative changes in the number of recorded species as the sample size increases within a specific environment. It helps to assess the total number of species in a given area and compare species diversity among different communities.

To estimate the effects of stochastic processes on the plant community assembly among vegetation types, Sloan et al. (2006) constructed an NCM, applying non-linear least-squares to generate the best fit between the frequency of species occurrence and their relative abundance. The R^2^ value indicates the goodness of fit of the model and is calculated with the “Östman’s method” (Östman et al., 2010). When R^2^ is close to 1, the community assembly is fully consistent with stochastic processes. When not describing the community composition, R^2^ can be ≤ 0. Here, Nm represented the relationship between the probability of occurrence and the relative abundance in the region (Chen et al., 2019; Mo et al., 2021).

All data were analyzed using “Hmisc”, “stats4”, “minpack.lm”, “vegan”, and other packages in R 4.2.2 (R Core Team, 2022).

## 3 Result

### 3.1 Species composition among vegetation types

**Figure 2:**
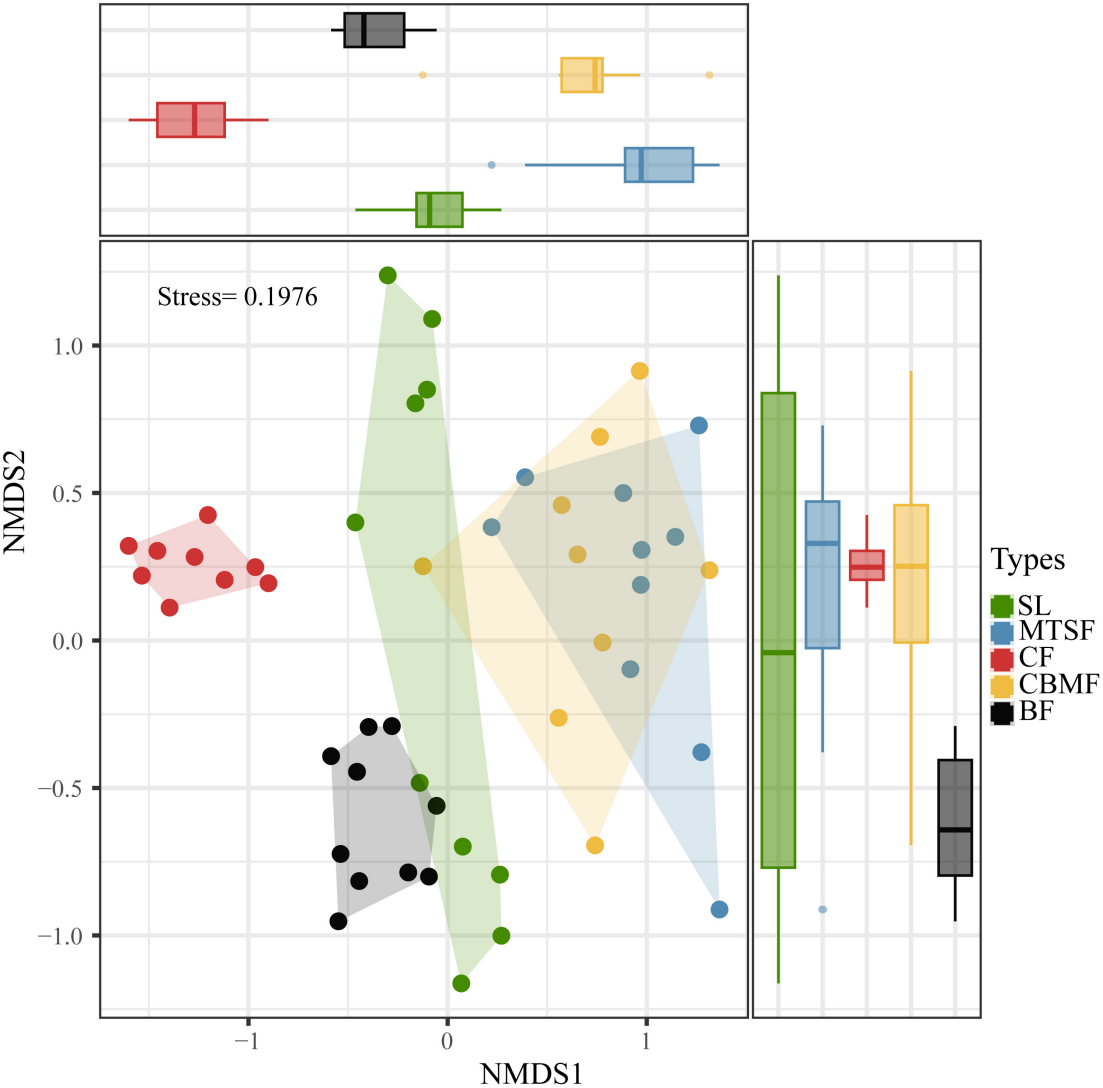
Non-metric multidimensional scaling (NMDS) ordination based on Bray-Curtis dissimilarity showing the community composition variations of the five vegetation types. SL, shrubland; MTSF, mixed tree and shrub forest; CF, coniferous forest; CBMF, coniferous broadleaf mixed forest; BF, broadleaf forest.

The NMDS show the stress is less than 2, the result is meaningful, furthermore significant differences between CF and other vegetation types on one axis, and its most concentrated on two-axis. Therefore, significant species composition differences between CF and other vegetation types, and the species composition of CBMF was similar to that of MTSF and significantly different from that of BF and CF. (**Figure 2**). The dominant species varies among vegetation types. SL is dominated by *C. parviflora*, with an IV of 6.92, while MTSF is dominated by *L. formosana*, with an IV of 10.27. As for CF, *P. kwangtungensis* is the dominant species, with an IV of 43.03, and *P. massoniana* dominates CBMF, with an IV of 23.73. A*. wangchii*, *B. minus* (6.03), and *Platycarya strobilacea* (4.69) are the dominant species in BF (**Supplementary Table S1**).

### 3.2 Species α-diversity index variations among vegetation types

**Figure 3:**
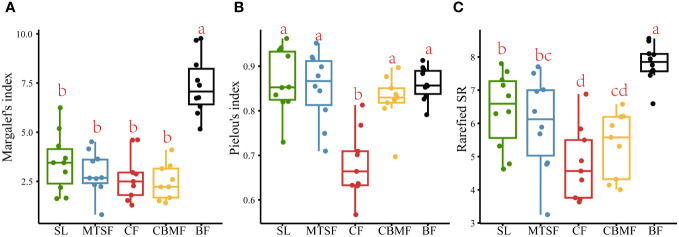
Variations in Margalef’s index of species richness **(A)**, Pielou’s index of species evenness **(B)**, and Rarefied species richness **(C)** among vegetation types. Multiple comparison tests were performed to determine the statistical significance, with *p* < 0.05 indicating significance. Scales labeled with identical letters have insignificant differences in their community feature values, and scales labeled with different letters have significant differences in their community feature values.

BF had the highest Margalef’s index of species richness among the five vegetation types, indicating the most species in plots. No significant variation was found among the other four vegetation types in Margalef’s index (**Figure 3A**). The Pielou’s index of species evenness of CF was significantly lower than those of the other four vegetation types, indicating a significant species dominance trend compared with other vegetation types (**Figure 3B**). The rarefied species richness results showed significant observed variations among all five vegetation types after controlling the confusing impacts of abundance. Besides, the BF still had the highest richness and significantly different from other vegetation types, and the CF is the lowest and significantly different from SL and MTSF (**Figure 3C**).

### 3.3 SADs and first-order assembly processes among vegetation types in karst landscape

**Figure 4:**
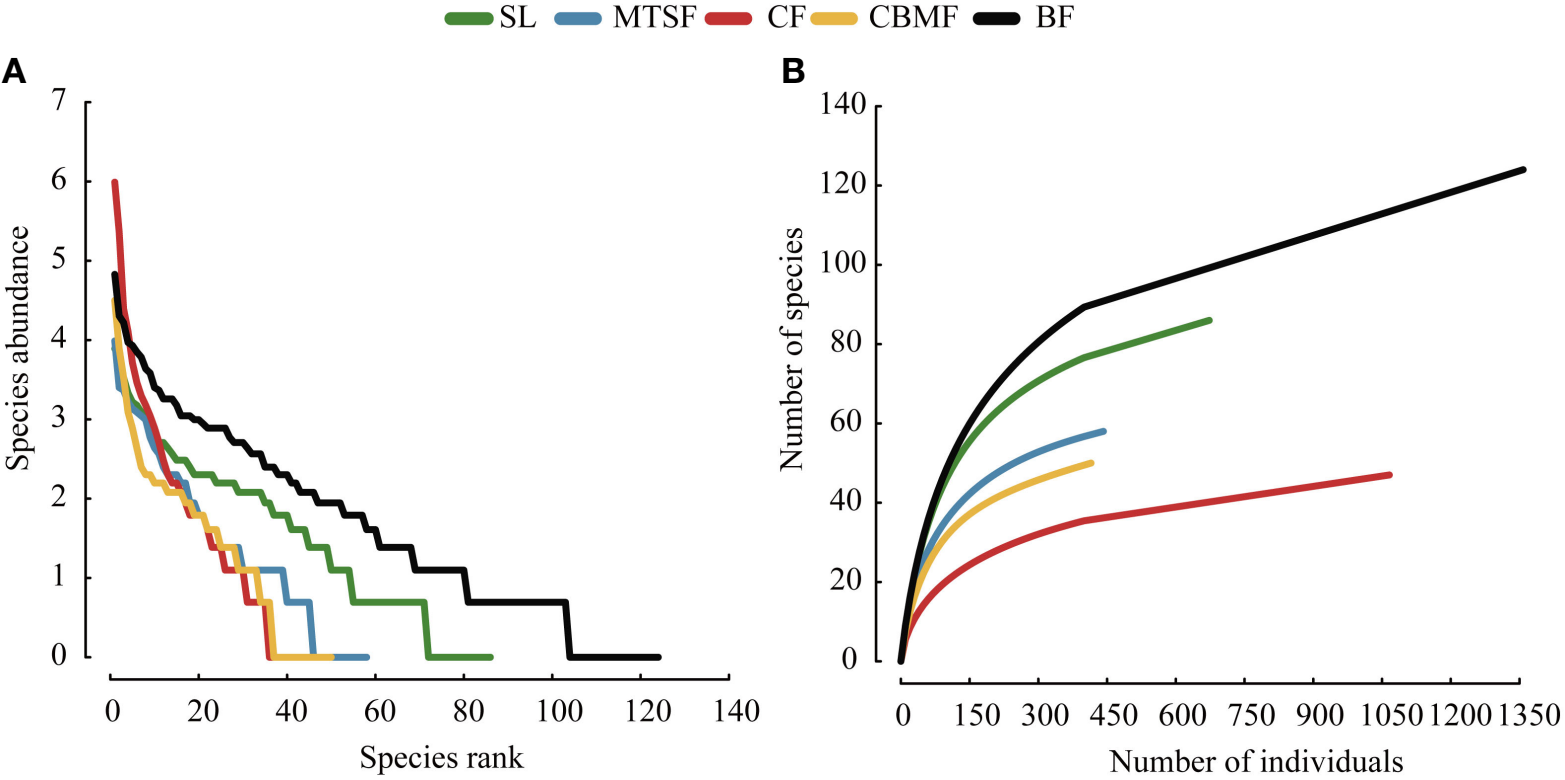
Species abundance distribution among vegetation types: **(A)** Species rank-abundance distribution curves. **(B)** Species-abundance accumulation curves.

CF has the highest decline rate and similar trends were observed on the species rank-abundance curves of MTSF and CBMF. BF and SL has slow decline rate on the species rank-abundance curves, which had lower species evenness than CF, MTSF and CBMF (**Figure 4A**). On the species accumulation curve, MTSF showed a similar trend to that of CBMF, and the species accumulation rate of CF was lower than other vegetation types and BF was largest (**Figure 4B**).

**Figure 5:**
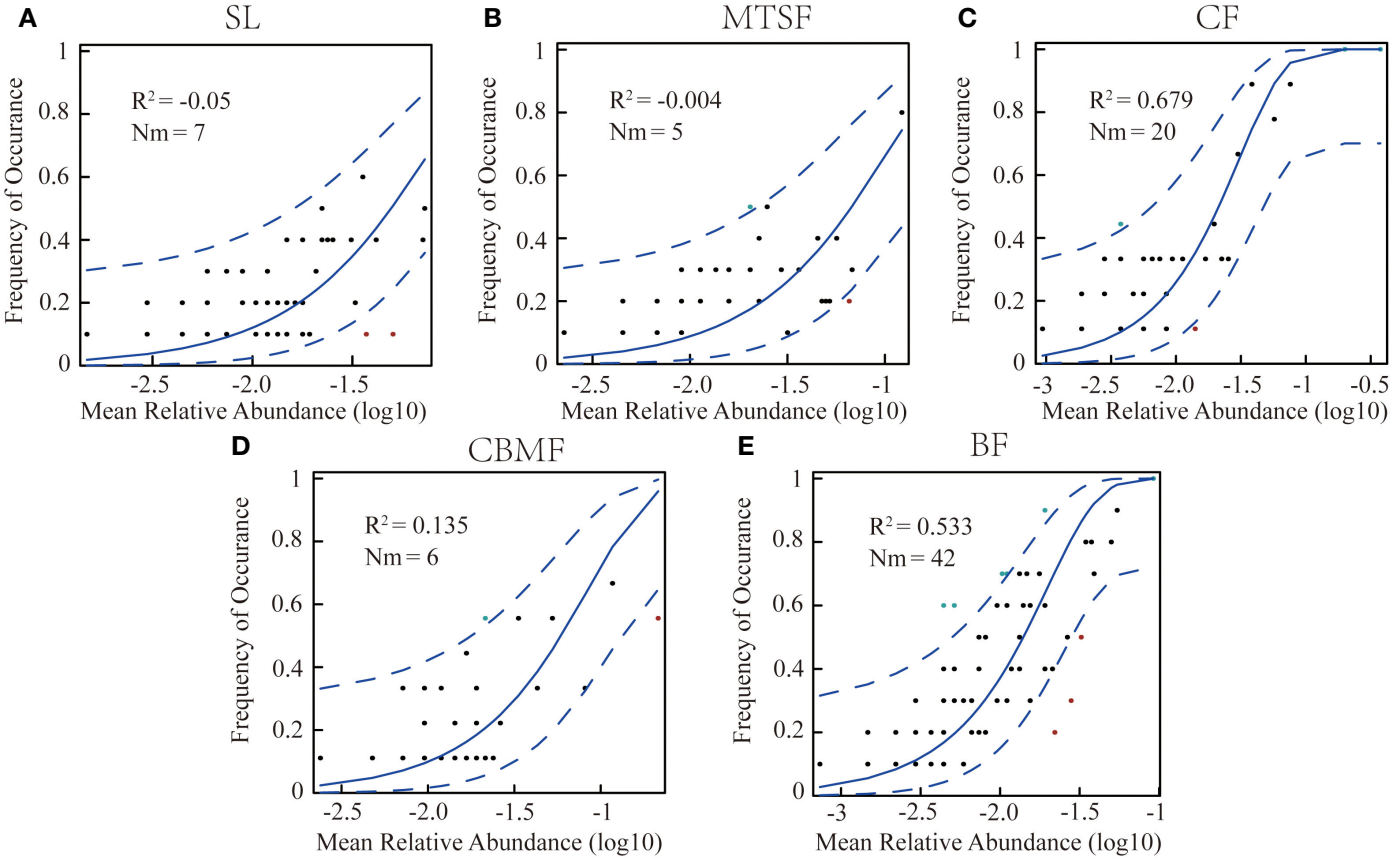
Fit of neutral community models(NCM) of community assembly among vegetation types. **(A)** NCM of shrubland. **(B)** NCM of mixed tree and shrub forest. **(C)** NCM of coniferous forest . **(D)** NCM of coniferous broadleaf mixed forest. **(E)** NCM of broadleaf forest. The predicted occurrence frequencies for the variations among vegetation types, plants, and communities, representing the differences in community ecological processes. The solid blue line is the best fit for the neutral community model (NCM) by Sloan et al. (2006), and the dashed blue line indicates 95% confidence intervals around the NCM prediction. Plants occurring more or less frequently than the NCM predictions are shown in green and red, respectively. *R^2^* represents the fit of this model. *Nm* indicates the community size times immigration.

The NCM results showed that the *R^2^* of CF (0.679) and BF (0.533) were significantly higher than the other three vegetation types, suggesting that the neutral process dominated the community assembly in BF and CF, while the niche process dominated the community assembly of the other three vegetation types (**Figure 5**). In addition, the highest *Nm* value in BF suggested a greater degree of species diffusion than other communities.

## 4 Discussion

This study compared the patterns of species diversity and potential community assembly of five vegetation types within the Maolan National Nature Reserve. The findings indicated that the CF community within the reserve exhibited strong uniqueness, with significant differences in species α-diversity and species composition. In terms of community assembly, significant differences were observed between the climax communities of CF and BF and the secondary SL, MTSF, and CBMF in the karst landscape.

### 4.1 Biodiversity patterns among vegetation types

As the comprehensive component of biodiversity, species composition can reflect the ecosystem structure and function (Rahman et al., 2022). Since the species diversity pattern involves the number of species and the species evenness, any changes in both components can lead to species composition variations (Chao et al., 2014). Our results showed significant variations among vegetation types in the karst landscape (**Figure 2**). Among all the five vegetation types, BF had the highest species richness, and CF had the lowest Pielou’s index of species evenness (**Figure 3**).

As a single dominant species in CF communities, *P. kwangtungensis* significantly controls the vegetation type structure and the community environment formation, with no obvious subdominant species and numerous rare species with low species evenness (**Figure 4A**; **Supplementary Table S1**). CF is normally distributed in habitats with higher altitudes or deeper slopes, where fewer species could colonize (Wang and Cui, 2023). Additionally, the aggregation strength of *P. kwangtungensis* increases with increasing elevation, thus occupying the niche space of other species. This could explain the lower species richness and evenness in CF than other vegetation types. On the other hand, the higher migration rate of rare species in CF inhibits the increase of species richness, leading to a pattern with a lower species richness and slower species accumulation rate (Stanley Harpole and Tilman, 2006; Xu et al., 2019). Thorn et al. (2020) found that rare species were the main factor of species composition differences. A higher rare species proportion in CF might be the main reason for its species composition variation compared with others (**Figure 2**). Consistent with the results of Zhang et al. (2012), our results showed that BF had the highest species richness and fastest species accumulation rate, with no obvious dominant species. BF in this study was established in subtropical evergreen deciduous broadleaf mixed forest, considered the climax in the karst landscape. Such a community normally features a complex community structure and a higher species richness (Qi et al., 2021). BF is used to considered in a relatively favorable environment. The relatively higher soil quality results in more individuals or abundance, possibly leading to a pattern with higher species richness (Zhang et al., 2022b). In addition, the stronger habitat heterogeneity at local scales supports various micro-habitats, which helps feed more species (Bátori et al., 2023). Thus, the relatively higher quality of habitat conditions and the stronger habitat heterogeneity might be the main reason for high species richness and evenness.

However, the species richness differed greatly among the five vegetation types after controlling the confusing impacts of abundance. A significant trend of BF > SL > MTSF > CBMF > CF was found in terms of rarefied species richness, which emphasized the importance of considering the impacts of sample size. In other words, individual variations among vegetation types could lead to a misunderstanding of species richness in the karst landscape. The species richness of SL was significantly higher than MTSF and CBMF after removing the influence of abundance on species richness (**Figure 3C**). In addition, the species richness of MTSF was significantly higher than that of CBMF, SL and MTSF are common secondary forests in karst areas, dominated by species such as *C. parviflora*, *L. formosana*, and *C. sinensis* (**Supplementary Table S1**). The lack of human disturbance allows shrubs to persist, with small-diameter tree species being the mainstay, leading to a scattered community advantage. Therefore, their species accumulation rates are second only to that of BF (**Figure 4B**), with high species evenness and no significant differences in species composition from other vegetation types except CF. Xiao et al. (2023) also suggested that high habitat heterogeneity of rocks increased shrub richness. In the MTSF community, tall deciduous trees gradually closed the canopy, limiting the understory light conditions (Li et al., 2016; Lu et al., 2016). Those heliophilous shrub species can not adapt to such poor light conditions and are excluded, thus exhibiting decreased species richness. However, the occurrence of tall deciduous trees facilitates the decomposition of fallen leaves and litter and promotes soil function recovery (Zeng et al., 2020). As a result, the immigration of shade-tolerant tree species, such as *Machilus nanmu*, *Quercus glauca*, and *Cornus kousa*, is promoted, leading to a higher species richness relative to CBMF. Like CF, CBMF was strongly dominated by extremely few species, such as *P. massoniana* and *L. formosana*. Such a monodominant community often leads to higher dominance and lower evenness. Consistent with the results of Zhang et al. (2022a) that *P. massoniana – L. formosana* mixed forest has higher species richness than CF, this study also showed a similar trend after controlling the sample size. The main reason is that adding broadleaf trees can increase the number of plant species and provide different types of litter for forest soil, thereby improving the physical and chemical properties of the soil (Zhang et al., 2022a). Moreover, adding broadleaf tree species also changes the resource acquisition strategies of the plants. Qin and Shangguan (2019) reported that compared with *P. massoniana* plantations, *P. massoniana – L. formosana* mixed forests show the most significant increase in leaf nutrient content and the best resource acquisition strategy to adapt to different environmental conditions.

### 4.2 Community assembly among vegetation types

The stochastic spread of species mainly depends on the difference between local species richness and diversity and the degree of diffusion restrictions. As a neutral-based process model, NCM is a valid approach for inferring stochastic processes acting on community assembly (Chen et al., 2019). This study used neutral models to simulate different vegetation types and found that both CF and BF communities were primarily governed by neutral processes, with CF being more strongly influenced by neutrality (R^2 ^= 0.68, explained by NCM), indicating that stochastic processes dominate the community assembly of CF (**Figure 5C**). The main reason is that in the CF community, *P. kwangtungensis* occupies an absolutely dominant position, with no obvious co-occurring species and relatively high rarity of other species (**Figure 4A**; **Supplementary Table S1**). In contrast, the species are more susceptible to stochastic processes due to their small population size, high migration rate, and limited geographical range (Xu et al., 2019; Zhang et al., 2022c). Additionally, the *P. kwangtungensis* population is large as it is adapted to high altitudes with low survival pressure. Coniferous trees often have allelopathic effects that inhibit the occurrence of other species and compress the ecological niche of other species within the community (Ehlers et al., 2014; Wang and Cui, 2023), resulting in strong randomness in species occurrence and stochastic processes dominating the community.

As the typical climax community in this landscape, BF communities have higher species richness and diversity than CF (**Figure 3**). Rare species are fewer within the communities, and the species distribution is relatively uniform. Therefore, the R^2^ value of the NCM is slightly lower than that of CF (**Figures 5C, E**). However, according to the calculated Nm values, species dispersal between plots in BF is higher than that in CF, indicating species dispersal as one of the important mechanisms of species coexistence in BF communities. Contradicting the findings of this research, however, Su et al. (2023) found that humidity and soil significantly impact species composition in karst BFs, indicating the effects of environmental filtering on ecological processes in the community. The reason may be that in the current study, dominant species in the karst BF community have adapted to the moisture stress and regulated their water use strategies by root plasticity and leaf shedding, adapting to the severe karstic habitat (Ding et al., 2023).

Previous studies suggested that secondary forests driven by deterministic processes could recover their community structure to the state before disturbance (Finegan, 1996; Letcher and Chazdon, 2009; Norden et al., 2009). In this study, the dominant ecological process in SL, MTSF, and CBMF tends to be the niche process, indicating that deterministic processes dominate the community assembly of these forests, which was consistent with the studies of tropical secondary forests by Norden et al. (2009), Cequinel et al. (2018) and others, where deterministic processes drove the composition of forest species. Previous studies have also reported that the colonization and extinction processes in forest communities are primarily determined by environmental heterogeneity and species competition (He et al., 2022). SL, MTSF, and CBMF are karstic secondary forest communities. The biological and non-biological filtering frameworks may be important in its ecological process (Münkemüller et al., 2020).

Despite the abundant rainfall in the karst landscape with a subtropical monsoon climate, the aboveground and underground dual-structure hydrological system often causes severe precipitation leakage, resulting in severe surface drought and frequent temporary droughts (Ding et al., 2023). Moreover, the karst surface is steep and irregular, with low and discontinuous soil coverage (Qi et al., 2021). Chase (2007) found that the community species composition similarities increased after drought treatment, possibly due to the strong drought-induced environmental filtering that filters out species with weak competitiveness and increases species similarity between communities. Therefore, environmental filtering may affect the construction of secondary forest communities. Among the three secondary forest communities, SL has the lowest R^2^ (**Figure 5A**), mainly because environmental filtering often has a smaller impact on larger species (Kristiansen et al., 2012). The SL communities are mostly dominated by small shrubs with a higher resource utilization efficiency than large trees and, therefore, a tendency to receive the most effects of ecological niche processes. As the typical dominant community in the karst landscapes, BF has high species diversities. Although CF communities’ species diversity is lower than BF communities, they are both dominant communities with stable community structures in karst areas. Thus, it can be indirectly inferred that the different developmental stages of karst communities may be governed by different community assembly mechanisms.

## 5 Conclusion

By quantifying the species diversity patterns and community assembly mechanisms among five vegetation types in the Maolan karst area, we found that the CF community had lower species evenness and richness, while the BF community had higher species richness. However, after controlling the sample size, a significant rarefied species richness trend of BF > SL > MTSF > CBMF > CF was found, which emphasized the importance of considering the confusing impacts of abundance. In addition, stochastic processes drove the community assembly of CF and BF, particularly species dispersal or formation, the climax communities in the karst landscape, while deterministic processes determined the community assembly of secondary communities such as SL, MTSF, or CBMF. At local scales, the difference among vegetation types was manifested in the potential community assembly rules in addition to the diversity patterns and community structure. Besides aboveground biomass and diversity, attention should also be directed to sorting ecological processes in regional forest ecology and management.

Our study provided clear evidence that the seeming different BF and CF were dominated by stochastic process, which emphasized the totally difference between climax communities and other communities. These findings improve understanding of species diversity patterns and community assembly rules in the karst landscape. However, we only examined the biodiversity patterns and community assembly rules at a given plot scale. Further study might benefit from performing relative study across spatial scales. In addition, further research also should consider more important explanatory deterministic factors and their relative importance such as unmeasured environmental conditions and species interactions. Furthermore, a focus should be placed on comparing the contributions of subterranean microorganisms in climax communities and other communities to the species diversity pattern and community assembly of above-ground vegetations communities.

## Data availability statement

The raw data supporting the conclusions of this article will be made available by the authors, without undue reservation.

## Author contributions

LM: Conceptualization, Methodology, Visualization, Writing – original draft. LZ: Funding acquisition, Methodology, Project administration, Supervision, Writing – review & editing. YL: Investigation, Writing – review & editing. LC: Investigation, Writing – review & editing. MS: Project administration, Supervision, Writing – review & editing. GZ: Project administration, Supervision, Writing – review & editing. QL: Project administration, Supervision, Writing – review & editing. DC: Project administration, Supervision, Writing – review & editing. YW: Investigation, Writing – review & editing. ZY: Investigation, Writing – review & editing. SC: Investigation, Writing – review & editing. RY: Resources, Writing – review & editing.
