# Supplementary material for: Variations in species diversity patterns and community assembly rules among vegetation types in the karst landscape
Source: Front Plant Sci. 2024 Feb 22;15:1338596. doi: 10.3389/fpls.2024.1338596 (PMC10917898; doi:10.3389/fpls.2024.1338596)
Supplement: Supplementary file 1 [file Table_1.docx]

Supplementary Material

# Supplementary Tables 1

IV of different vegetation types

| **Number** | **Vegetation types** | **Species name** | **Relative density**  **(%)** | **Relative significance (%)** | **Relative frequency (%)** | **Ⅳ (%)** |
| --- | --- | --- | --- | --- | --- | --- |
| 1 | SL | *Cornus parviflora* | 7.13 | 11.09 | 2.55 | 6.92 |
| 2 | SL | *Celtis sinensis* | 2.23 | 10.75 | 3.18 | 5.39 |
| 3 | SL | *Lindera communis* | 7.28 | 4.66 | 3.18 | 5.04 |
| 4 | SL | *Pteroceltis tatarinowii* | 2.38 | 6.93 | 2.55 | 3.95 |
| 5 | SL | *Rhus chinensis* | 3.57 | 3.59 | 3.82 | 3.66 |
| 6 | SL | *Ulmus pumila* | 1.19 | 7.25 | 1.91 | 3.45 |
| 7 | SL | *Tirpitzia sinensis* | 4.16 | 2.60 | 2.55 | 3.10 |
| 8 | SL | *Hovenia acerba* | 0.74 | 5.29 | 1.91 | 2.65 |
| 9 | SL | *Pyracantha fortuneana* | 2.53 | 2.31 | 2.55 | 2.46 |
| 10 | SL | *Handeliodendron bodinieri* | 1.49 | 4.55 | 1.27 | 2.44 |
| 11 | SL | *Symplocos sumuntia* | 3.12 | 1.60 | 2.55 | 2.42 |
| 12 | SL | *Camellia tuberculata* | 5.05 | 1.26 | 0.64 | 2.32 |
| 13 | SL | *Distylium racemosum* | 3.71 | 2.43 | 0.64 | 2.26 |
| 14 | SL | *Mallotus philippensis* | 1.78 | 2.28 | 2.55 | 2.20 |
| 15 | SL | *Machilus nanmu* | 0.45 | 4.88 | 1.27 | 2.20 |
| 16 | SL | *Ulmus castaneifolia* | 1.49 | 1.72 | 2.55 | 1.92 |
| 17 | SL | *Triadica rotundifolia* | 0.89 | 2.66 | 1.91 | 1.82 |
| 18 | SL | *Platycarya strobilacea* | 1.63 | 2.49 | 1.27 | 1.80 |
| 19 | SL | *Nandina domestica* | 2.23 | 0.35 | 2.55 | 1.71 |
| 20 | SL | *Ficus heteromorpha* | 1.34 | 2.47 | 1.27 | 1.69 |
| 21 | SL | *Viola collina* | 3.27 | 0.01 | 1.27 | 1.52 |
| 22 | SL | *Toxicodendron succedaneum* | 0.59 | 1.83 | 1.91 | 1.44 |
| 23 | SL | *Rubus biflorus* | 2.08 | 0.02 | 1.91 | 1.34 |
| 24 | SL | *Mahonia fortunei* | 1.19 | 0.39 | 1.91 | 1.16 |
| 25 | SL | *Mallotus repandus* | 1.78 | 0.18 | 1.27 | 1.08 |
| 26 | SL | *Myrsine semiserrata* | 1.93 | 0.64 | 0.64 | 1.07 |
| 27 | SL | *Litsea cubeba* | 0.89 | 1.00 | 1.27 | 1.05 |
| 28 | SL | *Picrasma quassioides* | 1.19 | 0.63 | 1.27 | 1.03 |
| 29 | SL | *Spiraea pubescens* | 1.34 | 0.48 | 1.27 | 1.03 |
| 30 | SL | *Mallotus tenuifolius* | 0.89 | 0.81 | 1.27 | 0.99 |
| 31 | SL | *Coriaria nepalensis* | 1.34 | 0.33 | 1.27 | 0.98 |
| 32 | SL | *Reevesia glaucophylla* | 1.49 | 0.80 | 0.64 | 0.97 |
| 33 | SL | *Hypericum monogynum* | 1.49 | 0.11 | 1.27 | 0.96 |
| 34 | SL | *Pterocarya orientalis* | 1.19 | 0.27 | 1.27 | 0.91 |
| 35 | SL | *Rosa cymosa* | 1.19 | 0.06 | 1.27 | 0.84 |
| 36 | SL | *Paederia foetida* | 1.78 | 0.09 | 0.64 | 0.84 |
| 37 | SL | *Cornus wilsoniana* | 0.89 | 0.33 | 1.27 | 0.83 |
| 38 | SL | *Viburnum dilatatum* | 1.04 | 0.13 | 1.27 | 0.81 |
| 39 | SL | *Boniodendron minus* | 1.34 | 0.44 | 0.64 | 0.80 |
| 40 | SL | *Camellia japonica* | 1.04 | 0.73 | 0.64 | 0.80 |
| 41 | SL | *Buddleja officinalis* | 1.49 | 0.20 | 0.64 | 0.77 |
| 42 | SL | *Viburnum fordiae* | 1.34 | 0.28 | 0.64 | 0.75 |
| 43 | SL | *Cinnamomum camphora* | 0.30 | 0.67 | 1.27 | 0.75 |
| 44 | SL | *Diospyros kaki* | 0.59 | 0.25 | 1.27 | 0.71 |
| 45 | SL | *Broussonetia papyrifera* | 0.30 | 1.09 | 0.64 | 0.68 |
| 46 | SL | *Urena lobata* | 1.19 | 0.04 | 0.64 | 0.62 |
| 47 | SL | *Ficus gaspariniana* | 0.45 | 0.10 | 1.27 | 0.61 |
| 48 | SL | *Machilus microcarpa* | 0.30 | 0.24 | 1.27 | 0.60 |
| 49 | SL | *Diospyros dumetorum* | 0.30 | 0.19 | 1.27 | 0.59 |
| 50 | SL | *Carpinus pubescens* | 0.74 | 0.38 | 0.64 | 0.59 |
| 51 | SL | *Phoebe crassipedicella* | 0.30 | 0.11 | 1.27 | 0.56 |
| 52 | SL | *Debregeasia orientalis* | 0.74 | 0.27 | 0.64 | 0.55 |
| 53 | SL | *Pistacia chinensis* | 0.30 | 0.58 | 0.64 | 0.51 |
| 54 | SL | *Machilus microphylla* | 0.30 | 0.50 | 0.64 | 0.48 |
| 55 | SL | *Carpinus turczaninowii* | 0.74 | 0.04 | 0.64 | 0.47 |
| 56 | SL | *Myrica rubra* | 0.45 | 0.31 | 0.64 | 0.46 |
| 57 | SL | *Carpinus lipoensis* | 0.59 | 0.08 | 0.64 | 0.44 |
| 58 | SL | *Ligustrum sinense* | 0.59 | 0.01 | 0.64 | 0.41 |
| 59 | SL | *Populus adenopoda* | 0.59 | 0.01 | 0.64 | 0.41 |
| 60 | SL | *Daphniphyllum oldhamii* | 0.15 | 0.45 | 0.64 | 0.41 |
| 61 | SL | *Spiraea blumei* | 0.45 | 0.12 | 0.64 | 0.40 |
| 62 | SL | *Zelkova serrata* | 0.30 | 0.26 | 0.64 | 0.40 |
| 63 | SL | *Euonymus nitidus* | 0.15 | 0.40 | 0.64 | 0.39 |
| 64 | SL | *Populus tomentosa* | 0.15 | 0.38 | 0.64 | 0.39 |
| 65 | SL | *Decaspermum gracilentum* | 0.45 | 0.06 | 0.64 | 0.38 |
| 66 | SL | *Sinoadina racemosa* | 0.30 | 0.19 | 0.64 | 0.37 |
| 67 | SL | *Viburnum foetidum* | 0.30 | 0.18 | 0.64 | 0.37 |
| 68 | SL | *Albizia kalkora* | 0.15 | 0.32 | 0.64 | 0.37 |
| 69 | SL | *Pittosporum glabratum* | 0.15 | 0.32 | 0.64 | 0.37 |
| 70 | SL | *Acer wangchii* | 0.30 | 0.16 | 0.64 | 0.37 |
| 71 | SL | *Fraxinus insularis* | 0.15 | 0.28 | 0.64 | 0.36 |
| 72 | SL | *Kalopanax septemlobus* | 0.30 | 0.10 | 0.64 | 0.34 |
| 73 | SL | *Mallotus barbatus* | 0.30 | 0.08 | 0.64 | 0.34 |
| 74 | SL | *Sloanea sinensis* | 0.30 | 0.08 | 0.64 | 0.34 |
| 75 | SL | *Clerodendrum cyrtophyllum* | 0.15 | 0.22 | 0.64 | 0.34 |
| 76 | SL | *Euscaphis japonica* | 0.30 | 0.02 | 0.64 | 0.32 |
| 77 | SL | *Vernicia fordii* | 0.30 | 0.02 | 0.64 | 0.32 |
| 78 | SL | *Lindera glauca* | 0.15 | 0.16 | 0.64 | 0.31 |
| 79 | SL | *Hylodesmum racemosum* | 0.30 | 0.00 | 0.64 | 0.31 |
| 80 | SL | *Liquidambar formosana* | 0.15 | 0.14 | 0.64 | 0.31 |
| 81 | SL | *Glochidion wilsonii* | 0.15 | 0.09 | 0.64 | 0.29 |
| 82 | SL | *Sorbus alnifolia* | 0.15 | 0.09 | 0.64 | 0.29 |
| 83 | SL | *Cinnamomum wilsonii* | 0.15 | 0.03 | 0.64 | 0.27 |
| 84 | SL | *Xylosma congesta* | 0.15 | 0.03 | 0.64 | 0.27 |
| 85 | SL | *Photinia bodinieri* | 0.15 | 0.01 | 0.64 | 0.27 |
| 86 | SL | *Ficus variegata* | 0.15 | 0.01 | 0.64 | 0.27 |
| 1 | MTSF | *Liquidambar formosana* | 12.22 | 11.81 | 6.78 | 10.27 |
| 2 | MTSF | *Castanopsis fargesii* | 4.75 | 18.68 | 1.69 | 8.38 |
| 3 | MTSF | *Cunninghamia lanceolata* | 6.79 | 10.18 | 2.54 | 6.50 |
| 4 | MTSF | *Ulmus pumila* | 5.66 | 9.28 | 3.39 | 6.11 |
| 5 | MTSF | *Celtis sinensis* | 4.52 | 4.77 | 3.39 | 4.23 |
| 6 | MTSF | *Koelreuteria paniculata* | 4.98 | 4.51 | 1.69 | 3.73 |
| 7 | MTSF | *Ficus variegata* | 6.56 | 1.89 | 1.69 | 3.38 |
| 8 | MTSF | *Quercus acutissima* | 5.20 | 2.40 | 1.69 | 3.10 |
| 9 | MTSF | *Diospyros kaki* | 2.49 | 1.96 | 4.24 | 2.89 |
| 10 | MTSF | *Populus przewalskii* | 2.94 | 2.74 | 2.54 | 2.74 |
| 11 | MTSF | *Broussonetia papyrifera* | 3.62 | 1.81 | 2.54 | 2.66 |
| 12 | MTSF | *Machilus nanmu* | 2.26 | 3.85 | 1.69 | 2.60 |
| 13 | MTSF | *Carpinus turczaninowii* | 0.68 | 5.55 | 0.85 | 2.36 |
| 14 | MTSF | *Lindera communis* | 2.04 | 0.46 | 4.24 | 2.24 |
| 15 | MTSF | *Cornus kousa* | 2.04 | 2.01 | 2.54 | 2.20 |
| 16 | MTSF | *Alangium chinense* | 2.26 | 0.62 | 3.39 | 2.09 |
| 17 | MTSF | *Albizia kalkora* | 1.58 | 0.94 | 2.54 | 1.69 |
| 18 | MTSF | *Platycarya strobilacea* | 2.26 | 1.01 | 1.69 | 1.66 |
| 19 | MTSF | *Emmenopterys henryi* | 3.17 | 0.69 | 0.85 | 1.57 |
| 20 | MTSF | *Pistacia chinensis* | 1.36 | 0.77 | 2.54 | 1.56 |
| 21 | MTSF | *Quercus glauca* | 0.90 | 1.21 | 2.54 | 1.55 |
| 22 | MTSF | *Prunus salicina* | 1.36 | 0.54 | 2.54 | 1.48 |
| 23 | MTSF | *Toxicodendron vernicifluum* | 0.68 | 1.88 | 1.69 | 1.42 |
| 24 | MTSF | *Ailanthus altissima* | 0.90 | 1.43 | 1.69 | 1.34 |
| 25 | MTSF | *Mallotus barbatus* | 1.13 | 0.32 | 2.54 | 1.33 |
| 26 | MTSF | *Ligustrum lucidum* | 1.58 | 0.45 | 1.69 | 1.24 |
| 27 | MTSF | *Morella rubra* | 1.13 | 0.63 | 1.69 | 1.15 |
| 28 | MTSF | *Platyosprion platycarpa* | 0.90 | 0.83 | 1.69 | 1.14 |
| 29 | MTSF | *Handeliodendron bodinieri* | 1.13 | 0.48 | 1.69 | 1.10 |
| 30 | MTSF | *Acer miyabei* | 0.68 | 0.75 | 1.69 | 1.04 |
| 31 | MTSF | *Sinoadina racemosa* | 0.90 | 0.28 | 1.69 | 0.96 |
| 32 | MTSF | *Vernicia fordii* | 0.68 | 0.48 | 1.69 | 0.95 |
| 33 | MTSF | *Rhamnus davurica* | 0.68 | 0.18 | 1.69 | 0.85 |
| 34 | MTSF | *Rhus chinensis* | 0.45 | 0.08 | 1.69 | 0.74 |
| 35 | MTSF | *Ligustrum sinense* | 0.45 | 0.08 | 1.69 | 0.74 |
| 36 | MTSF | *Triadica sebifera* | 0.90 | 0.34 | 0.85 | 0.70 |
| 37 | MTSF | *Cupressus funebris* | 0.23 | 1.00 | 0.85 | 0.69 |
| 38 | MTSF | *Coriaria nepalensis* | 0.68 | 0.25 | 0.85 | 0.59 |
| 39 | MTSF | *Tirpitzia sinensis* | 0.68 | 0.24 | 0.85 | 0.59 |
| 40 | MTSF | *Torricellia tiliifolia* | 0.68 | 0.18 | 0.85 | 0.57 |
| 41 | MTSF | *Quercus fabri* | 0.45 | 0.38 | 0.85 | 0.56 |
| 42 | MTSF | *Vitex cannabifolia* | 0.68 | 0.12 | 0.85 | 0.55 |
| 43 | MTSF | *Mallotus repandus* | 0.68 | 0.12 | 0.85 | 0.55 |
| 44 | MTSF | *Photinia davidsoniae* | 0.23 | 0.45 | 0.85 | 0.51 |
| 45 | MTSF | *Hovenia acerba* | 0.45 | 0.15 | 0.85 | 0.48 |
| 46 | MTSF | *Phoebe zhennan* | 0.45 | 0.11 | 0.85 | 0.47 |
| 47 | MTSF | *Betula luminifera* | 0.45 | 0.11 | 0.85 | 0.47 |
| 48 | MTSF | *Acer oblongum* | 0.23 | 0.28 | 0.85 | 0.45 |
| 49 | MTSF | *Prunus racemosa* | 0.23 | 0.28 | 0.85 | 0.45 |
| 50 | MTSF | *Symplocos sumuntia* | 0.23 | 0.14 | 0.85 | 0.40 |
| 51 | MTSF | *Rhamnus leptophylla* | 0.23 | 0.06 | 0.85 | 0.38 |
| 52 | MTSF | *Platycarya longipes* | 0.23 | 0.05 | 0.85 | 0.38 |
| 53 | MTSF | *Acer calcaratum* | 0.23 | 0.05 | 0.85 | 0.37 |
| 54 | MTSF | *Cornus controversum* | 0.23 | 0.05 | 0.85 | 0.37 |
| 55 | MTSF | *Pittosporum tobira* | 0.23 | 0.04 | 0.85 | 0.37 |
| 56 | MTSF | *Malus pumila* | 0.23 | 0.04 | 0.85 | 0.37 |
| 57 | MTSF | *Prunus pseudocerasus* | 0.23 | 0.04 | 0.85 | 0.37 |
| 58 | MTSF | *Prunus subg* | 0.23 | 0.00 | 0.85 | 0.36 |
| 1 | CF | *Pinus kwangtungensis* | 37.52 | 84.48 | 7.09 | 43.03 |
| 2 | CF | *Platycarya strobilacea* | 19.98 | 5.64 | 7.09 | 10.90 |
| 3 | CF | *Quercus phillyreoides* | 7.60 | 1.38 | 6.30 | 5.09 |
| 4 | CF | *Carpinus lipoensis* | 5.72 | 1.03 | 5.51 | 4.09 |
| 5 | CF | *Cyclobalanopsis glauca* | 3.85 | 0.82 | 6.30 | 3.65 |
| 6 | CF | *Acer sycopseoides* | 3.00 | 0.60 | 4.72 | 2.78 |
| 7 | CF | *Carpinus pubescens* | 2.53 | 0.98 | 2.36 | 1.96 |
| 8 | CF | *Cyclobalanopsis myrsinifolia* | 1.97 | 0.46 | 3.15 | 1.86 |
| 9 | CF | *Loropetalum chinense* | 2.25 | 0.34 | 2.36 | 1.65 |
| 10 | CF | *Castanopsis fargesii* | 1.69 | 0.52 | 2.36 | 1.52 |
| 11 | CF | *Boniodendron minius* | 1.13 | 0.39 | 2.36 | 1.29 |
| 12 | CF | *Pittosporum glabratum* | 0.38 | 0.07 | 3.15 | 1.20 |
| 13 | CF | *Michelia martini* | 0.94 | 0.22 | 2.36 | 1.17 |
| 14 | CF | *Photinia tushanensis* | 0.75 | 0.14 | 2.36 | 1.08 |
| 15 | CF | *Euscaphis japonica* | 0.66 | 0.15 | 2.36 | 1.06 |
| 16 | CF | *Ulmus castaneifolia* | 1.41 | 0.85 | 0.79 | 1.01 |
| 17 | CF | *Lithocarpus glaber* | 0.56 | 0.12 | 2.36 | 1.01 |
| 18 | CF | *Sinosideroxylon pedunculatum* | 0.56 | 0.10 | 2.36 | 1.01 |
| 19 | CF | *Triadica rotundifolia* | 0.38 | 0.22 | 2.36 | 0.99 |
| 20 | CF | *Cinnamomum camphora* | 0.28 | 0.09 | 2.36 | 0.91 |
| 21 | CF | *Tirpitzia sinensis* | 0.28 | 0.06 | 2.36 | 0.90 |
| 22 | CF | *Picrasma quassioides* | 0.84 | 0.13 | 1.57 | 0.85 |
| 23 | CF | *castanopsis fargesii* | 0.56 | 0.10 | 1.57 | 0.75 |
| 24 | CF | *Clausena dunniana* | 0.47 | 0.09 | 1.57 | 0.71 |
| 25 | CF | *Machilus microcarpa* | 0.28 | 0.09 | 1.57 | 0.65 |
| 26 | CF | *Itoa orientalis* | 0.28 | 0.08 | 1.57 | 0.64 |
| 27 | CF | *Calocedrus rupestris* | 0.28 | 0.04 | 1.57 | 0.63 |
| 28 | CF | *Elaeocarpus sylvestris* | 0.19 | 0.06 | 1.57 | 0.61 |
| 29 | CF | *Ficus gaspariniana* | 0.19 | 0.05 | 1.57 | 0.60 |
| 30 | CF | *Symplocos sumuntia* | 0.19 | 0.02 | 1.57 | 0.60 |
| 31 | CF | *Quercus fooningensis* | 0.84 | 0.13 | 0.79 | 0.59 |
| 32 | CF | *Machilus ichangensis* | 0.56 | 0.12 | 0.79 | 0.49 |
| 33 | CF | *Celtis sinensis* | 0.38 | 0.09 | 0.79 | 0.42 |
| 34 | CF | *Dendropanax dentiger* | 0.19 | 0.04 | 0.79 | 0.34 |
| 35 | CF | *Boniodendron minus* | 0.19 | 0.04 | 0.79 | 0.34 |
| 36 | CF | *Broussonetia papyrifera* | 0.09 | 0.04 | 0.79 | 0.31 |
| 37 | CF | *Pinus massoniana* | 0.09 | 0.03 | 0.79 | 0.31 |
| 38 | CF | *Toxicodendron vernicifluum* | 0.09 | 0.03 | 0.79 | 0.30 |
| 39 | CF | *Ficus microcarpa* | 0.09 | 0.03 | 0.79 | 0.30 |
| 40 | CF | *Ilex ficoidea* | 0.09 | 0.03 | 0.79 | 0.30 |
| 41 | CF | *Quercus jenseniana* | 0.09 | 0.02 | 0.79 | 0.30 |
| 42 | CF | *Taxus wallichiana* | 0.09 | 0.02 | 0.79 | 0.30 |
| 43 | CF | *Itea chinensis* | 0.09 | 0.02 | 0.79 | 0.30 |
| 44 | CF | *Sinoadina racemosa* | 0.09 | 0.02 | 0.79 | 0.30 |
| 45 | CF | *Brachystegia spp* | 0.09 | 0.02 | 0.79 | 0.30 |
| 46 | CF | *Meliosma henryi* | 0.09 | 0.01 | 0.79 | 0.30 |
| 47 | CF | *Elaeocarpus decipiens* | 0.09 | 0.01 | 0.79 | 0.30 |
| 1 | CBMF | *Pinus massoniana* | 21.69 | 44.19 | 5.32 | 23.73 |
| 2 | CBMF | *Liquidambar formosana* | 11.81 | 10.57 | 6.38 | 9.59 |
| 3 | CBMF | *Machilus nanmu* | 8.19 | 8.87 | 3.19 | 6.75 |
| 4 | CBMF | *Celtis sinensis* | 5.30 | 3.94 | 5.32 | 4.85 |
| 5 | CBMF | *Diospyros kaki* | 3.37 | 0.95 | 5.32 | 3.22 |
| 6 | CBMF | *Castanopsis fargesii* | 4.34 | 1.52 | 3.19 | 3.02 |
| 7 | CBMF | *Quercus acutissima* | 1.93 | 3.43 | 3.19 | 2.85 |
| 8 | CBMF | *Lindera communis* | 2.17 | 0.40 | 5.32 | 2.63 |
| 9 | CBMF | *Cunninghamia lanceolata* | 1.45 | 5.32 | 1.06 | 2.61 |
| 10 | CBMF | *Quercus glauca* | 1.69 | 0.83 | 4.26 | 2.26 |
| 11 | CBMF | *Castanea mollissima* | 1.93 | 2.69 | 2.13 | 2.25 |
| 12 | CBMF | *Choerospondias axillaris* | 1.93 | 3.71 | 1.06 | 2.23 |
| 13 | CBMF | *Morella rubra* | 1.93 | 0.80 | 3.19 | 1.97 |
| 14 | CBMF | *Symplocos sumuntia* | 2.65 | 0.79 | 2.13 | 1.86 |
| 15 | CBMF | *Vernicia fordii* | 1.20 | 0.59 | 3.19 | 1.66 |
| 16 | CBMF | *Carpinus pubescens* | 2.41 | 1.36 | 1.06 | 1.61 |
| 17 | CBMF | *Elaeocarpus decipiens* | 1.45 | 1.08 | 2.13 | 1.55 |
| 18 | CBMF | *Pistacia chinensis* | 0.96 | 0.32 | 3.19 | 1.49 |
| 19 | CBMF | *Ligustrum sinense* | 0.96 | 0.16 | 3.19 | 1.44 |
| 20 | CBMF | *Populus adenopoda* | 2.17 | 1.07 | 1.06 | 1.43 |
| 21 | CBMF | *Albizia kalkora* | 0.72 | 0.25 | 3.19 | 1.39 |
| 22 | CBMF | *Platycarya strobilacea* | 2.41 | 0.47 | 1.06 | 1.32 |
| 23 | CBMF | *Bauhinia purpurea* | 2.17 | 0.33 | 1.06 | 1.19 |
| 24 | CBMF | *Triadica rotundifolia* | 1.69 | 0.50 | 1.06 | 1.09 |
| 25 | CBMF | *Betula luminifera* | 0.96 | 0.12 | 2.13 | 1.07 |
| 26 | CBMF | *Bridelia tomentosa* | 0.72 | 1.05 | 1.06 | 0.95 |
| 27 | CBMF | *Toxicodendron vernicifluum* | 0.72 | 0.90 | 1.06 | 0.90 |
| 28 | CBMF | *Pteroceltis tatarinowii* | 1.45 | 0.15 | 1.06 | 0.89 |
| 29 | CBMF | *Photinia tushanensis* | 0.96 | 0.44 | 1.06 | 0.82 |
| 30 | CBMF | *Metapanax davidii* | 1.20 | 0.19 | 1.06 | 0.82 |
| 31 | CBMF | *Nandina domestica* | 1.20 | 0.15 | 1.06 | 0.81 |
| 32 | CBMF | *Triadica sebifera* | 0.48 | 0.49 | 1.06 | 0.68 |
| 33 | CBMF | *Platyosprion platycarpa* | 0.72 | 0.19 | 1.06 | 0.66 |
| 34 | CBMF | *Handeliodendron bodinieri* | 0.72 | 0.19 | 1.06 | 0.66 |
| 35 | CBMF | *Coriaria nepalensis* | 0.24 | 0.49 | 1.06 | 0.60 |
| 36 | CBMF | *Cornus controversum* | 0.48 | 0.22 | 1.06 | 0.59 |
| 37 | CBMF | *Camellia tuberculata* | 0.24 | 0.34 | 1.06 | 0.55 |
| 38 | CBMF | *Rhus chinensis* | 0.48 | 0.05 | 1.06 | 0.53 |
| 39 | CBMF | *Pittosporum tobira* | 0.24 | 0.18 | 1.06 | 0.50 |
| 40 | CBMF | *Broussonetia papyrifera* | 0.24 | 0.15 | 1.06 | 0.49 |
| 41 | CBMF | *Ulmus pumila* | 0.24 | 0.14 | 1.06 | 0.48 |
| 42 | CBMF | *Loropetalum chinense* | 0.24 | 0.12 | 1.06 | 0.48 |
| 43 | CBMF | *Cornus kousa* | 0.24 | 0.06 | 1.06 | 0.45 |
| 44 | CBMF | *Prunus salicina* | 0.24 | 0.06 | 1.06 | 0.45 |
| 45 | CBMF | *Cinnamomum wilsonii* | 0.24 | 0.03 | 1.06 | 0.44 |
| 46 | CBMF | *Cinnamomum camphora* | 0.24 | 0.03 | 1.06 | 0.44 |
| 47 | CBMF | *Ilex chinensis* | 0.24 | 0.02 | 1.06 | 0.44 |
| 48 | CBMF | *Kalopanax septemlobus* | 0.24 | 0.02 | 1.06 | 0.44 |
| 49 | CBMF | *Clausena dunniana* | 0.24 | 0.02 | 1.06 | 0.44 |
| 50 | CBMF | *Ficus variegata* | 0.24 | 0.02 | 1.06 | 0.44 |
| 1 | BF | *Acer wangchii* | 9.20 | 7.91 | 2.70 | 6.60 |
| 2 | BF | *Boniodendron minus* | 5.45 | 10.21 | 2.43 | 6.03 |
| 3 | BF | *Platycarya strobilacea* | 3.90 | 8.29 | 1.89 | 4.69 |
| 4 | BF | *Clausena dunniana* | 5.01 | 2.41 | 2.16 | 3.19 |
| 5 | BF | *Carpinus pubescens* | 1.33 | 5.83 | 1.08 | 2.75 |
| 6 | BF | *Cornus parviflora* | 3.76 | 2.15 | 2.16 | 2.69 |
| 7 | BF | *Pteroceltis tatarinowii* | 1.55 | 4.37 | 1.62 | 2.51 |
| 8 | BF | *Diospyros kaki* | 1.33 | 3.94 | 1.35 | 2.20 |
| 9 | BF | *Lindera communis* | 3.46 | 0.60 | 2.16 | 2.07 |
| 10 | BF | *Pistacia chinensis* | 1.33 | 2.95 | 1.89 | 2.05 |
| 11 | BF | *Machilus japonica* | 1.47 | 2.18 | 1.89 | 1.85 |
| 12 | BF | *Viburnum henryi* | 3.24 | 0.86 | 1.35 | 1.81 |
| 13 | BF | *Carpinus lipoensis* | 1.40 | 2.30 | 1.62 | 1.77 |
| 14 | BF | *Zelkova serrata* | 1.77 | 1.55 | 1.89 | 1.74 |
| 15 | BF | *Ulmus pumila* | 1.10 | 2.46 | 1.62 | 1.73 |
| 16 | BF | *Diospyros dumetorum* | 1.91 | 0.51 | 2.43 | 1.62 |
| 17 | BF | *Machilus microcarpa* | 1.10 | 2.74 | 0.81 | 1.55 |
| 18 | BF | *Euonymus laxiflorus* | 1.91 | 1.11 | 1.62 | 1.55 |
| 19 | BF | *Cornus wilsoniana* | 1.47 | 0.86 | 1.89 | 1.41 |
| 20 | BF | *Cinnamomum wilsonii* | 0.96 | 1.58 | 1.62 | 1.39 |
| 21 | BF | *Pittosporum glabratum* | 1.55 | 0.96 | 1.62 | 1.37 |
| 22 | BF | *Nandina domestica* | 2.65 | 0.07 | 1.35 | 1.36 |
| 23 | BF | *Toxicodendron succedaneum* | 1.10 | 1.05 | 1.89 | 1.35 |
| 24 | BF | *Handeliodendron bodinieri* | 0.96 | 1.23 | 1.62 | 1.27 |
| 25 | BF | *Triadica rotundifolia* | 0.52 | 1.59 | 1.62 | 1.24 |
| 26 | BF | *Castanopsis sclerophylla* | 2.80 | 0.11 | 0.81 | 1.24 |
| 27 | BF | *Miliusa balansae* | 2.14 | 0.29 | 1.08 | 1.17 |
| 28 | BF | *Euonymus nitidus* | 1.33 | 0.78 | 1.35 | 1.15 |
| 29 | BF | *Acer coriaceifolium* | 0.74 | 1.64 | 1.08 | 1.15 |
| 30 | BF | *Machilus microphylla* | 0.81 | 2.06 | 0.54 | 1.14 |
| 31 | BF | *Vitex canescens* | 0.52 | 2.01 | 0.81 | 1.11 |
| 32 | BF | *Quercus glauca* | 1.91 | 0.34 | 1.08 | 1.11 |
| 33 | BF | *Choerospondias axillaris* | 0.15 | 2.53 | 0.54 | 1.07 |
| 34 | BF | *Mallotus philippensis* | 1.03 | 0.25 | 1.89 | 1.06 |
| 35 | BF | *Gomphandra tetrandra* | 2.21 | 0.36 | 0.54 | 1.04 |
| 36 | BF | *Quercus thorelii* | 1.55 | 0.63 | 0.81 | 0.99 |
| 37 | BF | *Mahonia fortunei* | 1.33 | 0.11 | 1.35 | 0.93 |
| 38 | BF | *Elaeocarpus japonicus* | 0.81 | 0.61 | 1.35 | 0.92 |
| 39 | BF | *Celtis sinensis* | 0.81 | 0.61 | 1.35 | 0.92 |
| 40 | BF | *Carpinus londoniana* | 0.22 | 1.99 | 0.54 | 0.92 |
| 41 | BF | *Ficus variegata* | 0.74 | 0.65 | 1.35 | 0.91 |
| 42 | BF | *Lauro-cerasus spinulosa* | 0.52 | 1.36 | 0.81 | 0.90 |
| 43 | BF | *Viburnum propinquum* | 1.18 | 0.34 | 1.08 | 0.87 |
| 44 | BF | *Callicarpa giraldii* | 0.44 | 0.11 | 1.62 | 0.72 |
| 45 | BF | *Beilschmiedia kweichowensis* | 0.52 | 0.38 | 1.08 | 0.66 |
| 46 | BF | *Bennettiodendron leprosipes* | 0.96 | 0.13 | 0.81 | 0.63 |
| 47 | BF | *Fraxinus insularis* | 0.44 | 0.65 | 0.81 | 0.63 |
| 48 | BF | *Prunus mume* | 0.07 | 1.55 | 0.27 | 0.63 |
| 49 | BF | *Symplocos adenophylla* | 0.59 | 0.43 | 0.81 | 0.61 |
| 50 | BF | *Neolitsea confertifolia* | 0.59 | 0.41 | 0.81 | 0.60 |
| 51 | BF | *Quercus myrsinifolia* | 0.59 | 0.37 | 0.81 | 0.59 |
| 52 | BF | *Eurycorymbus cavaleriei* | 0.29 | 1.18 | 0.27 | 0.58 |
| 53 | BF | *Pittosporumcrispulum* | 0.66 | 0.21 | 0.81 | 0.56 |
| 54 | BF | *Croton tiglium* | 0.44 | 0.15 | 1.08 | 0.56 |
| 55 | BF | *Cinnamomum cassia* | 0.52 | 0.25 | 0.81 | 0.53 |
| 56 | BF | *Hovenia acerba* | 0.15 | 1.13 | 0.27 | 0.52 |
| 57 | BF | *Acer buergerianum* | 0.74 | 0.25 | 0.54 | 0.51 |
| 58 | BF | *Castanopsis fargesii* | 0.29 | 0.35 | 0.81 | 0.49 |
| 59 | BF | *Quercus jenseniana* | 0.52 | 0.04 | 0.81 | 0.45 |
| 60 | BF | *Photinia prunifolia* | 0.44 | 0.36 | 0.54 | 0.45 |
| 61 | BF | *Distylium tsiangii* | 0.66 | 0.12 | 0.54 | 0.44 |
| 62 | BF | *Dendropanax dentiger* | 0.29 | 0.11 | 0.81 | 0.40 |
| 63 | BF | *Eriobotrya japonica* | 0.29 | 0.04 | 0.81 | 0.38 |
| 64 | BF | *Tirpitzia sinensis* | 0.37 | 0.23 | 0.54 | 0.38 |
| 65 | BF | *Litsea pungens* | 0.22 | 0.29 | 0.54 | 0.35 |
| 66 | BF | *Cinnamomum camphora* | 0.29 | 0.17 | 0.54 | 0.34 |
| 67 | BF | *Viburnum dilatatum* | 0.59 | 0.14 | 0.27 | 0.33 |
| 68 | BF | *Ulmus castaneifolia* | 0.29 | 0.15 | 0.54 | 0.33 |
| 69 | BF | *Ilex pentagona* | 0.22 | 0.20 | 0.54 | 0.32 |
| 70 | BF | *Grona heterocarpos* | 0.22 | 0.47 | 0.27 | 0.32 |
| 71 | BF | *Rhapis excelsa* | 0.37 | 0.02 | 0.54 | 0.31 |
| 72 | BF | *Machilus nanmu* | 0.29 | 0.09 | 0.54 | 0.31 |
| 73 | BF | *Morus liboensis* | 0.15 | 0.22 | 0.54 | 0.30 |
| 74 | BF | *Viburnum brachybotryum* | 0.44 | 0.15 | 0.27 | 0.29 |
| 75 | BF | *Bridelia retusa* | 0.22 | 0.10 | 0.54 | 0.29 |
| 76 | BF | *Kalopanax septemlobus* | 0.15 | 0.17 | 0.54 | 0.29 |
| 77 | BF | *Sloanea sinensis* | 0.37 | 0.21 | 0.27 | 0.28 |
| 78 | BF | *Vitex negundo* | 0.22 | 0.09 | 0.54 | 0.28 |
| 79 | BF | *Machilus ichangensis* | 0.22 | 0.09 | 0.54 | 0.28 |
| 80 | BF | *Euonymus alatus* | 0.22 | 0.07 | 0.54 | 0.28 |
| 81 | BF | *Sinoadina racemosa* | 0.22 | 0.04 | 0.54 | 0.27 |
| 82 | BF | *Euonymus dielsianus* | 0.15 | 0.09 | 0.54 | 0.26 |
| 83 | BF | *Distylium myricoides* | 0.22 | 0.01 | 0.54 | 0.26 |
| 84 | BF | *Dalbergiahupeana* | 0.15 | 0.35 | 0.27 | 0.25 |
| 85 | BF | *Firmiana simplex* | 0.15 | 0.06 | 0.54 | 0.25 |
| 86 | BF | *Toona sinensis* | 0.15 | 0.06 | 0.54 | 0.25 |
| 87 | BF | *Cornus elliptica* | 0.22 | 0.22 | 0.27 | 0.24 |
| 88 | BF | *Ilex latifolia* | 0.15 | 0.02 | 0.54 | 0.23 |
| 89 | BF | *Coriaria nepalensis* | 0.15 | 0.01 | 0.54 | 0.23 |
| 90 | BF | *Murraya exotica* | 0.15 | 0.01 | 0.54 | 0.23 |
| 91 | BF | *Albizia kalkora* | 0.15 | 0.01 | 0.54 | 0.23 |
| 92 | BF | *Carpinus turczaninowii* | 0.07 | 0.33 | 0.27 | 0.23 |
| 93 | BF | *Lindera pulcherrima* | 0.15 | 0.24 | 0.27 | 0.22 |
| 94 | BF | *Lauro-cerasus zippeliana* | 0.15 | 0.24 | 0.27 | 0.22 |
| 95 | BF | *Buxus megistophylla* | 0.29 | 0.04 | 0.27 | 0.20 |
| 96 | BF | *Metadina trichotoma* | 0.15 | 0.11 | 0.27 | 0.18 |
| 97 | BF | *Morus alba* | 0.22 | 0.01 | 0.27 | 0.17 |
| 98 | BF | *Aesculus chinensis* | 0.15 | 0.08 | 0.27 | 0.17 |
| 99 | BF | *Distylium racemosum* | 0.15 | 0.07 | 0.27 | 0.16 |
| 100 | BF | *Buxus henryi* | 0.07 | 0.11 | 0.27 | 0.15 |
| 101 | BF | *Sinosideroxylon pedunculatum* | 0.07 | 0.10 | 0.27 | 0.15 |
| 102 | BF | *Pyracantha loureiroi* | 0.15 | 0.03 | 0.27 | 0.15 |
| 103 | BF | *Ligustrum expansum* | 0.07 | 0.10 | 0.27 | 0.15 |
| 104 | BF | *Acersinense* | 0.15 | 0.02 | 0.27 | 0.15 |
| 105 | BF | *Ilex chinensis* | 0.15 | 0.01 | 0.27 | 0.14 |
| 106 | BF | *Miliusasinensis* | 0.15 | 0.01 | 0.27 | 0.14 |
| 107 | BF | *Photinia beauverdiana* | 0.15 | 0.00 | 0.27 | 0.14 |
| 108 | BF | *Myrsine semiserrata* | 0.15 | 0.00 | 0.27 | 0.14 |
| 109 | BF | *Reevesia glaucophylla* | 0.07 | 0.03 | 0.27 | 0.13 |
| 110 | BF | *Tarennoidea wallichii* | 0.07 | 0.03 | 0.27 | 0.12 |
| 111 | BF | *Cleidion bracteosum* | 0.07 | 0.03 | 0.27 | 0.12 |
| 112 | BF | *Machilus rehderi* | 0.07 | 0.02 | 0.27 | 0.12 |
| 113 | BF | *Acerlongipes* | 0.07 | 0.02 | 0.27 | 0.12 |
| 114 | BF | *Litsea coreana* | 0.07 | 0.01 | 0.27 | 0.12 |
| 115 | BF | *Styrax japonicus* | 0.07 | 0.01 | 0.27 | 0.12 |
| 116 | BF | *Xylosma congesta* | 0.07 | 0.01 | 0.27 | 0.12 |
| 117 | BF | *Pyracantha fortuneana* | 0.07 | 0.01 | 0.27 | 0.12 |
| 118 | BF | *Ilex macrocarpa* | 0.07 | 0.01 | 0.27 | 0.12 |
| 119 | BF | *Callicarpa bodinieri* | 0.07 | 0.01 | 0.27 | 0.12 |
| 120 | BF | *Gleditsia sinensis* | 0.07 | 0.00 | 0.27 | 0.12 |
| 121 | BF | *Debregeasia orientalis* | 0.07 | 0.00 | 0.27 | 0.12 |
| 122 | BF | *Rhamnus napalensis* | 0.07 | 0.00 | 0.27 | 0.12 |
| 123 | BF | *Osmanthus yunnanensis* | 0.07 | 0.00 | 0.27 | 0.11 |
| 124 | BF | *Bischofia javanica* | 0.07 | 0.00 | 0.27 | 0.11 |
